# Kramecyne — A New Anti-inflammatory Compound Isolated from *Krameria cytisoides*

**DOI:** 10.3390/molecules17022049

**Published:** 2012-02-20

**Authors:** Salud Pérez-Gutiérrez, Ernesto Sánchez-Mendoza, Diana Martínez-González, Miguel Angel Zavala-Sánchez, Cuauhtemoc Pérez-González

**Affiliations:** Departamento de Sistemas Biológicos, Universidad Autónoma Metropolitana-Xochimilco, Calzada del Hueso 1100, Col. Villa Quietud, Coyoacan C.P. 04960, D.F.A.P. 23-181, Mexico

**Keywords:** anti-inflammatory activity, *Krameria*, peroxide, polymer, kramecyne

## Abstract

In the present work we describe the structure and anti-inflammatory activity of a new compound, kramecyne, isolated from a methanol extract of *Krameria cytisoides* (Krameriaceae). The structure of kramecyne was determined by IR, NMR, MS, and elemental analysis, which indicated that the structure corresponded to a hexamer of cyclic peroxide monomers. This compound exhibited good anti-inflammatory activity in the 12-*O*-tetradecanoylphorbol-13-acetate (TPA)-induced mouse ear edema (51.8 ± 6.9% inhibition) and carrageenan-induced rat paw edema models at doses of 50 mg/kg. The compound significantly reduced edema to 63.1% after 1.0 h, and the effect was unchanged for 5 h. Kramecyne did not present acute toxicity, even at doses of 5,000 mg/kg.

## 1. Introduction

Inflammatory diseases are an important cause of human mortality and morbidity. Many diseases are associated with inflammation, including infections by bacteria, viruses, and protozoa, and autoimmune diseases, such as arthritis, diabetes, Alzheimer’s, and cancer. Inflammation is typically treated using non-steroidal anti-inflammatory drugs (NSAIDs) and corticosteroids, but the secondary effects can be significant.

Many plants have been used in traditional medicine to prevent or minimize the progression of inflammation. *Krameria cytisoides* Cav (Krameriaceae) is a perennial erect shrub found across the Americas. It is typically up to 1.8 m in height, obovate leaves up to 2 cm long, purplish flowers, 2 cm long sepals. The plant is used in Mexican traditional medicine to treat diarrhea, hemorrhoids, sore throat, sore gums, and sore nipples [1], and it is effective for inflammations of the genitalia. It is also taken for urinary tract problems and painful rectal conditions [2], and the root is used to treat stomach and bowel cancer inflammatory diseases [3]. Twenty-one lignans, neolignans, and norneolignans have been isolated from the roots [4], but the anti-inflammatory activities of this plant and its extracts have not previously been examined.

In this study we reported the structure of a cyclic peroxide named kramecyne which has been isolated for the first time from the methanol extract of *K. cytisoides*; the anti-inflammatory properties of this compound were also tested on two models *in vivo* and acute toxicity was determined.

## 2. Results and Discussion

The optical rotation of kramecyne was [α]^20°^ = 36.9°, and the IR data were (ν, cm^−1^ solid): 3,332 (O-H), 2,919 and 2,486 (C-H), 1,608 (skeleton), 1,443 (C-C), 1,284, 1,108 (C-O), and 800 (O-O) [5]. The molecular weight was obtained from a positive FAB-MS, which indicated protonated molecular [M+H]^+^ ions at *m/z* 793, [(M/2)+H]^+^ at 397, and [(M/6)+4H]^+^ at 136. Elemental analysis showed that the molecule contained C 45.23, H 6.02, O 48.23%. The combined molecular weight and elemental analysis data provided a molecular formula of C_30_H_48_O_24_.

### 2.1. NMR Studies

^1^H, ^13^C, DEPT, gCOSY, gHSQC, and gHMBC NMR spectra were recorded in a methanol-d_4_ solution, and all NMR data are reported in Table 1. The NMR-^1^H spectrum showed a signal overlap at δ = 3.58 and 3.82 ppm, and the chemical shifts were typical of aliphatic protons vicinal to the oxygen [6]. The ^13^C-NMR spectrum presented five signals at δ_13C_ 62.12, 62.85, 63.40, 73.17, and 75.15 ppm. These chemical shifts confirmed that each carbon was bonded to oxygen.

**Table 1:** NMR spectroscopy data for kramecyne (500 MHz, methanol-d_4_).

Position	δ_C_, type	δ_Ha_, mult(*J* in Hz)	δ_Hb_, mult(*J* in Hz)
3	63.40, CH_2_	3.61, d (11.47)	3.63, d (11.47)
4	75.15, C	-	-
5	73.17, CH	3.74, dd (-, 3.19)	-
6	62.12, CH_2_	3.69, m	3.78, dd (10.54, 3.19)
7	62.85, CH_2_	3.63, d (11.50)	3.67, d (11.50)

**Figure 1:**
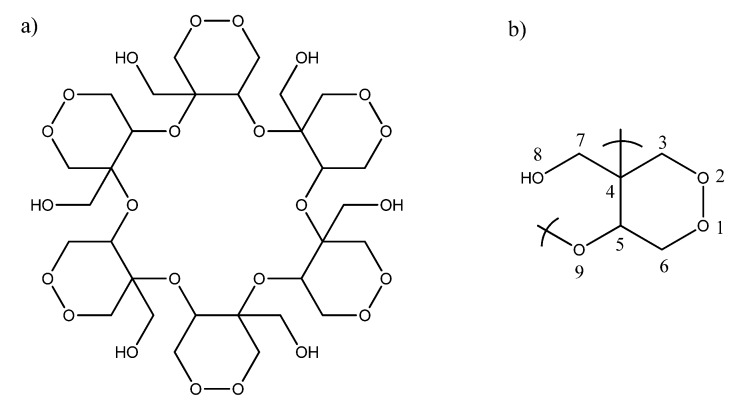
(**a**) Kramecyne (cyclic polymer); (**b**) Monomer.

The number of peaks in the ^13^C-NMR spectrum and the integration values in the ^1^H-NMR spectra suggested that the molecule was highly symmetric. According to the molecular weight and the number of carbons, we propose that the C_30_H_48_O_24_ molecule is a cyclic polymer (Figure 1a) composed of six monomers of formula C_5_H_8_O_6_ (Figure 1b). The monomers are linked by formation of a cyclic ether between atoms 4–9 [6].

The DEPT spectrum was helpful in the elucidation the types of C atoms and showed that each monomer included three CH_2_, one CH, and one quaternary carbon. Each monomer was a cyclic peroxide with a hydroxymethylene and an ether groups. The correlations between protons and carbons were assigned based on an analysis of the gCOSY and gHSQC NMR spectra, the values of which are reported in Table 1. The structural position of the quaternary carbon was confirmed from the gHMBC NMR correlations.

**Figure 2:**
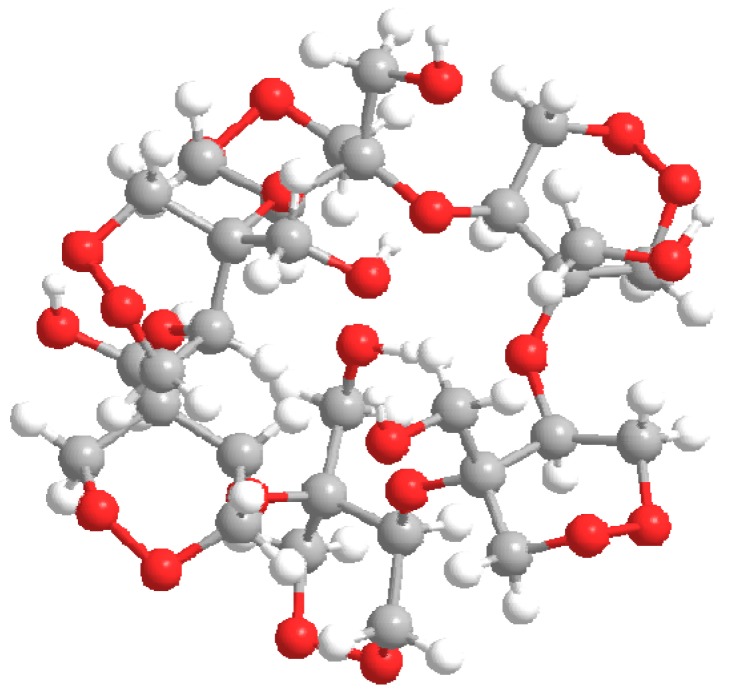
Kramecyne. The 3D structure was optimized at the B3LYP/6-31G level of theory.

The three-dimensional structure of kramecyne (Figure 2) was optimized using a DFT approach [7] with the B3LYP/6-31G method level of approximation. The vibrational frequencies were real, indicating that the structure assumed a minimum energy conformation [8]. The optimized geometry was used to predict the theoretical ^1^H-NMR and ^13^C-NMR spectra. The spectra calculated using the ACD Labs package agreed well with the experimental data (see Table 1). The ^13^C-NMR spectrum included peaks at δ 64.10, 65.10, 68.05, 76.69, and 80.08 ppm. The chemical shifts of the protons were predicted from theory to occur between δ 3.55 and δ 4.35 ppm. A comparison between the ^1^H-NMR experimental and calculated spectra revealed the same signal patterns.

**Figure 3:**
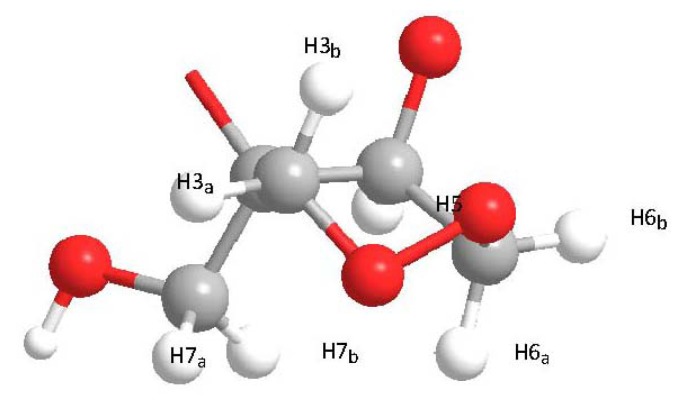
3D structure of kramecyne monomer.

Figure 3 shows a three-dimensional optimized structure of the monomer, in which the H3 and H6 methylene protons were present in the equatorial and axial conformations. This conformation was well-defined by the conformational restraints of the cyclic peroxide. The H7 protons were not equivalent because they could not be interchanged by a symmetry operation in any conformation [7]. HSQC experiments confirmed that the methylene protons were diastereotopic and, therefore, provided different chemical shifts.

### 2.2. Kramecyne Acetate

The ^1^H-NMR and ^13^C-NMR spectra showed that the O-O bond of the peroxide reacts with the Ac_2_O, resulting in the formation of two additional acetic ester moieties and the third acetate group by the hydroxyl group reaction. The polymeric cyclic structure is maintained during the acetylation reaction and included 18 acetate groups, which is confirmed by the APCI-MS spectrum that showed *m/z* = 1,566.6, corresponding to [M+H], but due to the symmetry of this structure the ^13^C-NMR spectrum only showed three signals of carbonyl groups.

The ^13^C-NMR spectrum presented three carbonyl groups at δ 169.54, 170.29, and 170.50 ppm, and the carbons of the monomer have signals at δ 62.02, 64.15, 64.35, 71.15, and 73.45 ppm. The ^1^H-NMR spectrum also confirmed the successful acetylation. All monomer signals were deshielded by the three ester groups, the protons of which yielded peaks at δ 4.18, 4.55, and 5.30 ppm, and the three methyl groups, with peaks at δ 2.1, 2.2, and 2.3 ppm.

### 2.3. Anti-inflammatory Activity

The anti-inflammatory activity of chloroform and methanol extracts on TPA-induced ear edema was tested next; the chloroform extract did not show any activity on this model, so we followed the study with the methanol extract, which had a significant effect (50.7 ± 3.2% inhibition).

The anti-inflammatory activity of kramecyne on TPA-induced ear edema was also tested. At a dose of 2 mg/ear, this compound showed significant activity (51.8 ± 6.9% inhibition), similar to that obtained with indomethacin (64.8 ± 5.8% inhibition). The pronounced inflammation induced by TPA when is administered topically is thought to be mediated by protein kinase C, and the stimulation of phospholipase A_2_, and cyclooxygenase [10], which results in the release of arachidonic acid and prostaglandin E_2_ [11]. Thus, both phospholipase A_2_ and cyclooxygenase inhibitors are active in this model for inflammation, and kramecyne may interfere with these mediators to inhibit TPA-induced inflammation.

Kramecyne, tested *in vivo*, showed significant activity on carrageenan-induced inflammation relative to the control group (Table 2). This compound had no activity at doses of 200 and 400 mg/kg 4 h after carrageenan injection. However, at a dose of 50 mg/kg, kramecyne significantly reduced inflammation by 63.1% after 1 h, and the effect was unchanged during the following 5 h. At this dose, kramecyne showed the best effects, and at dose of 25 mg/kg showed a similar effect to indomethacin after 5 h, although with this dose there is no activity after 1 h. Two hours after administering at a dose of 100 mg/kg, the inflammation was reduced to 70.1%, and after 3 h the inhibition was 46.9%, the inhibition effect of kramecyne is not dose dependent (Table 2). Although the indomethacin dose is lower than that of kramecyne; the LD_50_ of indomethacin in rats is 13 mg/kg [12], while it was not possible to determine the LD_50_ of kramecyne because this compound has no toxicity at a dosis of 5,000 mg/kg.

**Table 2:** Anti-inflammatory activity of kramecyne on carrageenan-induced edema.

Time h	Indomethacin	(% inhibition)				
		Kramecyne				
	8 mg/kg	25 mg/kg	50 mg/kg	100 mg/kg	200 mg/kg	400 mg/kg
1	26.4 ± 2.1 *	17.5 ± 13.04	63.1 ± 12.1 *	52.2 ± 6.1 *	58.6 ± 6.4 *	42.5 ± 11.1 *
2	30.0 ± 4.5 *	30.8 ± 8.24	55.7 ± 5.6 *	70.1 ± 5.7 *	36.9 ± 6.3 *	41.7 ± 6.8 *
3	63.1 ± 7.1 *	40.5 ± 5.54	51.4 ± 5.5 *	46.9 ± 5.8 *	29.9 ± 4.6	33.1 ± 5.9
4	68.3 ± 8.1 *	46.1 ± 4.05 *	56.4 ± 4.2 *	43.2 ± 2.7	24.9 ± 5.5	28.6 ± 5.0
5	67.6 ± 6.6 *	60.1 ± 4.09 *	64.5 ± 4.1 *	45.2 ± 5.1	21.2 ± 4.9	19.9 ± 2.7

Results are expressed as percentage of inhibition and mean of eight determinations ± SE. ANOVA one way, Tuckey test * *p* < 0.05 for comparison of kramecine with indomethacin treated groups.

Leukocyte migration to an injured tissue is an important aspect of the inflammatory process. The release of several mediators of the phlogistic response, including histamine and serotonin, are responsible for the immediate inflammation response [13], whereas kinins and prostaglandins mediate the prolonged response [14] On the other hand, some plant constituents significantly inhibit the biosynthetic pathways of inflammation mediators [14]. Kramecyne showed effects against this model, suggesting that its anti-inflammatory activity was possibly due to inhibition of the production of these mediators.

Kramecyne did not present acute toxicity, even at the highest studied doses (5,000 mg/kg). No damage in the liver, lung, heart and kidney was observed. Kramecyne thus presents very low toxicity in the model test, but it is necessary to carry out more experiments for chronic toxicity evaluation. This low toxicity opens the possibility of using this compound in the treatment of inflammatory diseases. Experiments are in progress to further evaluate the molecular mechanism through which kramecyne exerts its anti-inflammatory activity.

## 3. Experimental

### 3.1. General

The mass spectrum was recorded using the Fast Atom Bombardment (FAB) ionization method in the positive mode by direct injection on a Jeol-102 mass spectrophotometer, using a nitrobenzyl alcohol-like matrix. Elemental analysis was conducted using an Exeter Analytical Inc. CE-440 elemental analyzer, and acetanilide was used as the reference compound. The IR spectrum was recorded in the solid phase using a Perkin-Elmer Paragon 1000 FT-IR spectrophotometer. All NMR spectra were recorded at 298 K on a Bruker Avance DMX500 spectrometer operated at 500.13309 MHz for ^1^H-NMR and 125.77036 MHz for ^13^C-NMR. ^1^H- and ^13^C-NMR chemical shifts were reported relative to TMS and CDCl_3_, respectively, and compound was dissolved in methanol-d_4_. The DEPT, gCOSY, gHSQC, and gHMBC spectra were measured using the standard Bruker pulse sequences. The kramecyne acetate was dissolved in CDCl_3_, and the chemical shifts were reported with respect to TMS and CDCl_3_.

### 3.2. Plant Material

*K. cytisoides* was collected in Las Comadres Municipality of Guadalcazar, San Luis Potosi State, Mexico, in June 2009. The taxonomic identification of the plant was confirmed by taxonomist José García Pérez. A voucher specimen (SPLM44560) was deposited in the Isidro Palacios Herbarium of the Universidad Autónoma de San Luis Potosí.

### 3.3. Isolation of Kramecyne

The shade-dried leaves of *K. cytisoides* were reduced to powder, then a portion (200 g) were defatted with hexane (2 L) at boiling point for 4 h, and then the material was extracted into MeOH (2 L) under reflux for 4 h. The methanol extract was concentrated to half the original volume under reduced pressure, and a dark brown solid was obtained in 3% yield (m.p. 172 °C, dec.). The compound’s purity was determined by thin-layer chromatography, ^1^H-NMR and ^13^C-NMR spectra.

### 3.4. Kramecyne Acetate

A solution of kramecyne (100 mg) and pyridine (2 mL) was acetylated with Ac_2_O (3.5 mL) at room temperature overnight. After the usual workup, the crude product was purified by silica gel chromatography. The triacetate obtained had a mp of 125–127 °C. 

### 3.5. Detection of Peroxides

Twenty milligrams of the compound were dissolved in methanol, a few drops of a 5% potassium iodide solution were added, and the solution was shaken. The appearance of a yellow-to-brown color indicated the presence of peroxides [15].

### 3.6. Animals

Male Wistar Rats (150–200 g) and male mice of the CD1 strain (20–25 g) from the Universidad Autónoma Metropolitana-Xochimilco animal facility were housed in isolated cages at 24 °C under a light-dark cycle of 12:12. The animals were supplied with food (Purina) and water *ad libitum*.

### 3.7. 12-O-Tetradecanoylphorbol-13-acetate (TPA)-Induced Mouse Ear Edema

The model for TPA-induced edema in mouse ears has been described previously [16]. A solution containing TPA (2.5 μg) in acetone (25 μL) was topically applied to the inner and outer surfaces of the right ears of male CD1 mice, and acetone alone was applied to the inner and outer surfaces on the left ear. Thirty minutes thereafter, kramecyne or indomethacin (2.0 mg) dissolved in acetone were topically applied to the right ear and acetone was applied to the left ear six hours later, the animals were sacrificed, and 6 mm plugs of the central portion of both ears were weighed. The percentage inhibition of edema was determined.

### 3.8. Carrageenan an Induced Rat Paw Edema

Paw edema was induced by intradermal injection of 0.1 mL of a 1% carrageenan suspension in the left hind foot pad. One hour prior to carrageenan injection, groups of eight rats each were treated with 25, 50, 100, 200, or 400 mg/kg kramecyne. The control rats received the vehicle alone (polyvinyl pyrrolidone, PVP) and the reference group received 8 mg/kg indomethacin, the administration was orally. The paw volume was measured by the volume displacement method using a plethysmometer (Ugo Basile) 1.0, 2.0, 3.0, 4.0, and 5.0 h after carrageenan administration. The percentage of edema inhibition was determined [17].

### 3.9. Acute Toxicity

Kramecyne was orally administered as a single dose to groups of mice (n = 3) at different concentrations (1,000–5,000 mg/kg). The dose ranges used in mice followed the method of Lorke [17]. After administration, the animals were observed under open-field conditions for a 72 h period; the animals were sacrificed and were observed damage of heart, lung, liver and kidney. The number of animal deaths and signs of clinical toxicity were recorded in this period.

### 3.10. Statistical Analysis

Data are expressed as mean ± S.E.M. Statistical analysis was performed using the Student’s t-test (P < 0.05), while ANOVA followed by Tukey test P < 0.05 were considered as indicative of significance.

## 4. Conclusions

From the methanol extract of *K. cytisoides* a new cyclic peroxide composed of six monomers was obtained. The structure was characterized by different spectroscopical methods. Kramecyne had anti-inflammatory activity in the acute inflammation models used in this work when administered topical and orally, and it showed low acute toxicity.
